# The high burden of erectile dysfunction among men living with HIV in northern Tanzania: a call for evidence-based interventions

**DOI:** 10.3389/fruro.2023.1238293

**Published:** 2023-09-13

**Authors:** Orgeness J. Mbwambo, Moses Lyatuu, Geofrey Ngocho, Khadija Abdallah, Patricia Godfrey, Bartholomeo N. Ngowi, Alex Mremi, Evangelista Malindisa, Maryam Amour, James Ngocho, Emmanuel Balandya, Gideon Kwesigabo, Rachel Manongi, Benson R. Kidenya, Stephen E. Mshana, Eligius F. Lyamuya, Bruno F. Sunguya, John Bartlett, Blandina Theophil Mmbaga, Alfred K. Mteta

**Affiliations:** ^1^ Faculty of Medicine, Kilimanjaro Christian Medical University College, Moshi, Tanzania; ^2^ Department of Urology, Kilimanjaro Christian Medical Centre, Moshi, Tanzania; ^3^ Department of Pathology, Kilimanjaro Christian Medical Centre, Moshi, Tanzania; ^4^ Department of Physiology, Catholic University of Health and Allied Sciences, Moshi, Tanzania; ^5^ Department of Internal Medicine, Muhimbili University of Health and Allied Sciences, Dar es Salaam, Tanzania; ^6^ Department of Physiology, Muhimbili University of Health and Allied Sciences, Dar es Salaam, Tanzania; ^7^ Department of Epidemiology and Biostatistics, Muhimbili University of Health and Allied Sciences, Dar es Salaam, Tanzania; ^8^ Institute of Public Health, Kilimanjaro Christian Medical University College, Moshi, Tanzania; ^9^ Deaprtment of Biochemistry and Molecular Biology, Catholic University of Health and Allied Sciences, Mwanza, Tanzania; ^10^ Department of Microbiology, Catholic University of Health and Allied Sciences, Mwanza, Tanzania; ^11^ Department of Microbiology, Muhimbili University of Health and Allied Sciences, Dar es Salaam, Tanzania; ^12^ Department of Public Health, Muhimbili University of Health and Allied Sciences, Dar es Salaam, Tanzania; ^13^ Global Health and Nursing, Duke University, Durham, NC, United States; ^14^ Kilimanjaro Christian Research Institute, Moshi, Tanzania

**Keywords:** men living with HIV, erectile dysfunction, Tanzania, antiretroviral, risk factors

## Abstract

**Background:**

The extent of the burden of erectile dysfunction and its associated factors remains unclear. The aim of this study was to investigate the factors associated with ED and its prevalence among MLHIV in northern Tanzania.

**Methods:**

A hospital-based, multi-center, cross-sectional study was conducted on MLHIV aged 18 years and above in northern Tanzania.

**Outcome:**

The risk factors for ED and the prevalence of such risk factors among MLHIV was assessed and evaluated through a multivariate logistic regression analysis adjusted for depression symptoms using the Patient Health Questionnaire-9 (PHQ9) scale; anxiety disorders using the Generalized Anxiety Disorder Assessment (GAD-7); ART adherence; viral load; initial regimen date; ART regimen; and sexual risk behaviors.

**Results:**

Data for 366 participants with a median age of 50 years (IQR 38–57 years) were available for analysis. Approximately three in four (74.6%) MLHIV had ED (of any severity), whereas 37.7% had mild ED. The majority (96.5%) of the participants had low testosterone, two in three (66.7%) had depressive symptoms, and close to half of the participants (48.4%) had anxiety. Age, lack of engagement in vigorous physical activity, depression, and self-reported good adherence to antiretroviral therapy (ART) were associated with ED in a multivariate logistic regression analysis (*p*=0.004, *p* =0.006, *p*=0.07, *p*=0.006, and *p*=0.004, respectively).

**Conclusion:**

There is a high prevalence of ED among MLHIV in northern Tanzania. Erectile dysfunction should be regarded as one of the comorbidities associated with HIV and should be routinely screened for among MLHIV in CTC clinics.

## Introduction

Erectile dysfunction (ED) is defined as the persistent inability to achieve and maintain an erection for adequate to carry out satisfactory sexual acts (1, 2). The burden of ED is commonly higher among people with chronic diseases, including men living with human immunodeficiency virus (MLHIV) (3–6). The burden of ED in MLHIV is higher than that in the general population and ED also remains the most common sexual dysfunction in MLHIV (6, 7). The risk of acquiring ED is 2.3 times higher in HIV patients than in people without HIV (7, 8). Evidence suggests that prevalent of ED in MLHIV ranges between 30 to 50% even in men under 40 years. This suggests that there could be additional factors causing the rate of ED to be higher among people with HIV than in the general population.

Possible factors contributing to the higher prevalence of ED among MLHIV include some ARV drug groups, such as the protease inhibitors ritonavir and saquinavir; hypogonadism; an increase in life expectancy in HIV patients; non-communicable diseases, such as type 2 diabetes mellitus, depression, and dyslipidemia; and the effect of HIV (6–9). However, the evidence remains controversial, indicating a multifactorial etiology for ED (10).

Sexual problems, including ED, if overlooked and under-managed in HIV patients, are known to impair the quality of life and general health of patients, interfering with intimate relationships and lowering adherence to antiretroviral medications (6, 11–14). Evidence has shown an association between ED and an increase in risky sexual behavior, decreased adherence to antiretroviral drug regimens, an increased risk of trans-mission of drug-resistant strains because of high- risk sexual behavior, and higher HIV RNA concentrations in semen (3, 7, 15). A study conducted in Tanzania in 2013 suggested a high rate of sexual risk behaviors among HIV- infected young males (16). Risky sexual behavior and poor adherence to ART represent a challenge to the management of HIV and achievement of the 95–95–95 target, and have been linked to ED among MLHIV. In northern Tanzania, the most common ART regimen used comprises tenofovir, lamivudine, and dolutegravir.

Despite the fact that ED is more prevalent in HIV patients than in the general population, little attention has been paid internationally to the diagnosis and management of ED in HIV patients, and Tanzania is no exception. Little or no evidence is available about such burdens in countries such as Tanzania. Even in Western countries, where some studies have been conducted, the association between HIV and ED remains inconclusive. This study, therefore, aims to determine the prevalence of and factors associated with erectile dysfunction among men living in northern Tanzania.

## Methods

### Study area and population

A cross-sectional design was used in this multi-center study to examine the prevalence of and factors associated with erectile dysfunction among MLHIV in the Moshi municipality of the Kilimanjaro region in Tanzania from April 2022 to September 2022. The four care and treatment centers (CTCs) where this study was conducted were the Kilimanjaro Christian Medical Center (KCMC), a zonal tertiary hospital; the Mawenzi regional referral hospital; and the Majengo and Pasua health centers, all of which are located in the Moshi municipality. These facilities were selected due to the volume of patients. The study population was male patients who attended the outpatient clinics in the four CTCs in the Kilimanjaro region aged 18 years and above who consented to be part of the research.

### Inclusion and exclusion criteria

Adult MLHIV aged 18 years and above attending CTC who consented to participate were included in the study. Patients who had not been sexually active for more than 6 months were excluded.

#### Sample size and sampling

The sample size for this study was estimated using the Leslie Kish formula (*n*=z^2^p(100-p/ε^2^), where *n* was the estimated minimum sample size, Z was the confidence level at 95% (standard value is 1.96), p was the prevalence of ED in a population-based study conducted in Nigeria (17) in which the prevalence of ED was 37.8%, and ϵ was the precision at 95% CI = 0.05. For this study, a minimum sample size of 361 was required. The convenience sampling technique was used to recruit participants.

### Study procedure and sample collection

Following participants’ completion of an informed consent form, a structured questionnaire was used for data collection, with both closed and open-ended questions to retrieve participant’s characteristics. Trained research assistants collected data through face-to-face interviews using a standardized questionnaire. The tool included social demographic variables, namely, erectile dysfunction assessment using the International Index of Erectile Function: (IIEF-5); depression symptoms, using the Patient Health questionnaire-9 scale (PHQ9); and anxiety disorders, using the Generalized Anxiety Disorder Assessment (GAD-7). Clinical data, such as those related to ART adherence, viral load, initial regimen date, and ART regimen, were retrieved from patients’ files. Adherence was assessed using data from the pharmacy. The tool also included questions on risky sexual behaviors, which were unprotected sexual intercourse, oral sex, anal sex, and having more than one sexual partner. Data pertaining to patients’ body mass index (BMI) and random blood sugar (RBG), testosterone, cholesterol, and triglyceride levels were retrieved from the results of laboratory investigations.

### Quality control

Standard operating procedures were observed during the process of data collection and analysis. A MAGLUMI machine was used for hormonal analysis (of estrogen and testosterone) and BioSystem was used for lipid analysis (of cholesterol and triglyceride).

### Data management and analysis

Data were analyzed using Stata SE, version 15.0. For descriptive statistics, results were expressed in tables, graphs, and pie charts. ED was categorized as mild, moderate, or severe according to the IIEF-5 (1–7: severe ED, 8–11: moderate ED, 12–16: mild-to-moderate ED, and 17–21: mild ED). Hypertension was defined as a blood pressure > 130/90 mmHg, and was measured twice at intervals of 5 min. Those with a random blood sugar level of > 11 mmol/L or current history of taking antidiabetic drugs were considered patients with diabetes mellitus. ART adherence was classified as good (95% and above) for patients who scored all the four questions (i.e., did not miss any drug doses in the last 6 months) or poor (less than 95%) for participants who missed at least one question (i.e. missed a drug dose within the last 6 months).

The associations between categorical variables and erectile dysfunction were compared using a chi-square test. Univariate and multivariate logistic regression analyses were conducted for the risk factors of erectile dysfunction. Multivariate analysis was conducted only for those factors that were statistically significant in the univariate analysis.

### Ethical consideration

Ethical approval was obtained from the Kilimanjaro Christian Medical University College (KCMUCo) institution ethical review board (certificate number 2547). Permission to conduct the study was sought from the directors of the respective health facilities. All study participants were given a written informed consent form prior to participating in the interview. No information was shared with any third party, and numbers were used in the questionnaires instead of personal identifiers. Participants diagnosed with ED were referred to specialized departments for treatment.

## Results

### Sociodemographic characteristics of study participants

A total of 370 participants were enrolled in this study; however, the final analysis included 366 participants with a median age of 50 years (interquartile range 38 to 57 years). Four patients were excluded from the final analysis as they had not engaged in sexual intercourse for more than 6 months. The majority (55.2%) of participants were aged between 40 and 59 years, were married (58.7%), and had been educated to a primary level (58.2%). Among the 366 patients, only 21.8% were on a low income (i.e., earning less than 1 US dollar per day), almost half consumed alcohol (46.5%), and 17.8% were smokers or had a history of smoking. More than half of the participants (68.1%) engaged in vigorous physical activity, and half of the participants were engaged in risky sexual behavior (**Table 1**).

**Table 1:** Sociodemographic characteristics of the participants (*n*=366).

Characteristics	*n*	%
Age (years)		
18–39	94	25.7
40–59	202	55.2
≥60	70	19.1
Marital status		
Married	215	58.7
Cohabiting	3	0.8
Divorced/separated	40	10.9
Widower	25	6.8
Education level		
No formal education	12	3.2
Primary education	213	58.2
Secondary education	108	29.1
Higher education	33	9.0
Income		
Low income	80	21.8
High Income	286	78.2
Alcohol		
No	196	53.5
Yes	170	46.5
Smoking		
No	301	83.2
Yes	65	17.8
Engage in vigorous physical activity		
No	115	31.9
Yes	251	68.1
Engage in risky sexual behavior		
No	188	51.4
Yes	178	48.6

### Clinical characteristics

The majority of the participants had depression (66.7%), with half of the participants experiencing anxiety (48.4%). The majority of the patients had normal RBG levels (90.7%), and most were not overweight or obese (73.8%), with 16.4% having HTN. Almost all (96.5%) of the participants had low serum testosterone levels, and the majority had normal levels of estradiol, triglyceride, and cholesterol (83.9%, 79%, and 85.8%, respectively). Half of the participants had used ART for more than 5 years (69.9%), of which the majority had good self-reported adherence (87.7%). Most of the participants (92.6%) were taking first- line ART drugs (tenofovir, lamivudine, and dolutegravir) and most had a CD4 count > 200 (88.2%) (**Table 2**).

**Table 2:** Clinical characteristics of study participants (*n*=366).

Variable	*n*	%
Depression score		
No depression	122	33.3
Mild	183	50.0
Moderate	46	12.6
Moderate to severe	7	1.9
Severe	8	2.2
Anxiety score		
No anxiety	189	51.6
Mild	148	39.6
Moderate	21	5.7
Moderate to severe	7	1.9
Severe	4	1.1
Random blood glucose		
Normal RBG	332	90.7
High RBG	34	9.3
Hypertension		
No	306	83.6
Yes	60	16.4
BMI categories		
Normal/underweight	270	73.8
Overweight/obesity	96	26.2
Testosterone levels		
Low	353	96.5
High	13	3.5
Estradiol level		
Normal	307	83.9
High	59	16.1
Triglyceride level		
Normal	289	79.0
High	77	21.0
Cholesterol level		
Normal	314	85.8
High	52	14.2
ART adherence		
Good	321	87.7
Poor	45	12.3
Duration of ART		
1–5 years	110	30.1
6–10 years	110	30.1
>10 years	146	39.8
Type of ART drugs		
1RA	339	92.6
Other types of ART drugs	27	7.4
CD4 count		
≤200	43	11.8
>200	323	88.2

ART, anti-retroviral therapy; BMI, body mass index; 1RA, tenofovir, lamivudine, and dolutegravir; RBG, random blood glucose.

*1UA, zidovudine, lamivudine, and dolutegravir. 1PA, abacavir, lamivudine, and dolutegravir. 2KA, abacavir, lamivudine, and atazanavir/ritonavir. 2HA, tenofovir, emtricitabine, and atazanavir/ritonavir. 2GA, tenofovir, lamivudine, and efavirenz.

### Prevalence of erectile dysfunction in men living with HIV

**Figure 1:**
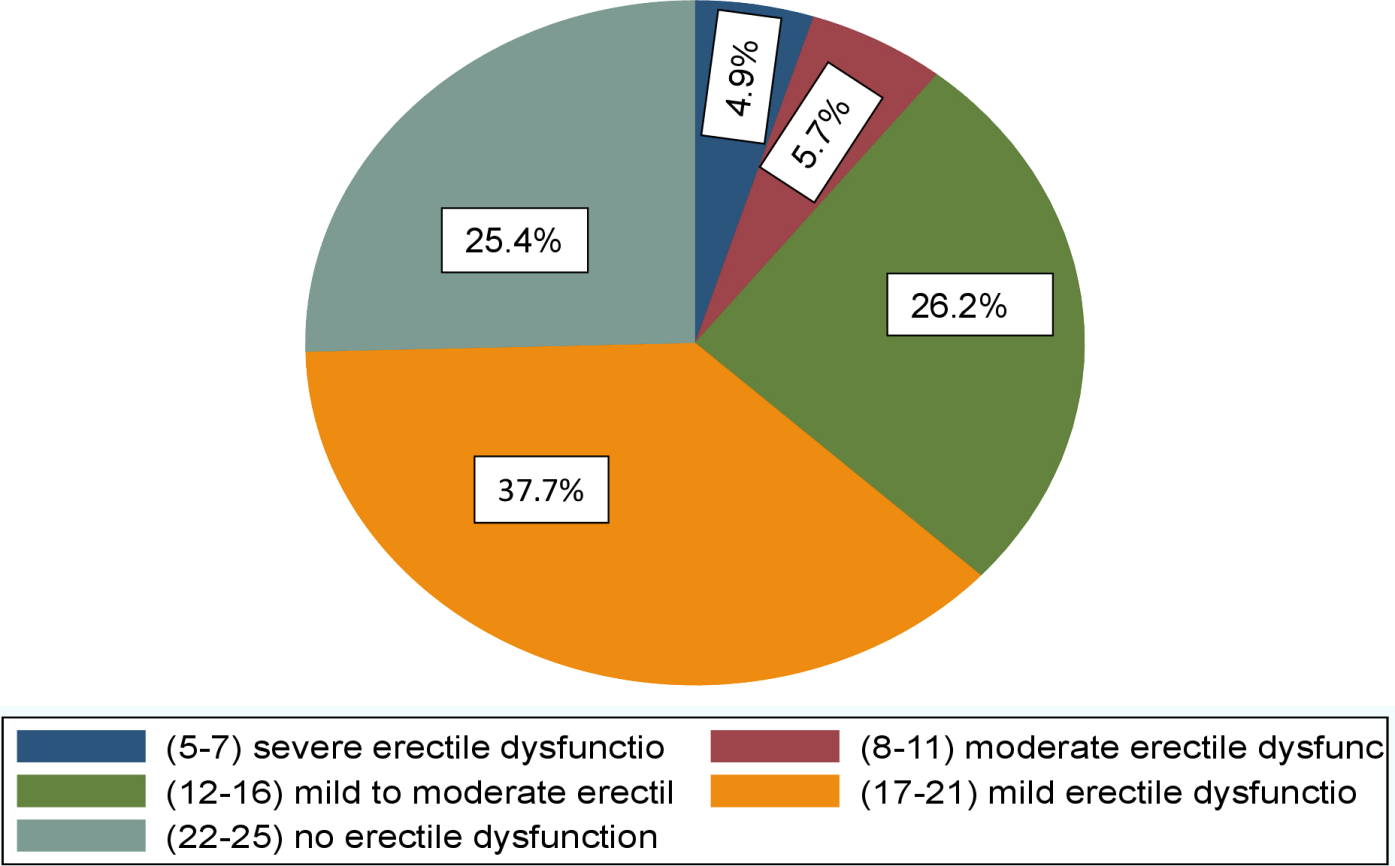
Prevalence of Erectile Dysfunction in men living with HIV in Northen Tanzania.

Three- quarters (74.6%) of the participants had ED, of which 37.7% had mild ED, 26.2% had mild-to-moderate ED, 5.7% had moderate ED, and 5.1% had severe ED (**Figure 1**).

### Participants characteristics associated with erectile dysfunction

The odds of developing ED in MLHIV at age 40 to 59 years were 2.9 times higher than for those aged <40 years in our multivariate analysis [*p* = 0.003; odds ratio (OR) =3.93; 95% confidence interval (CI) =1.45 to 5.86], and 7 times higher than for those aged >60 years (*p*<0.001; OR=2.55 to 19.60). For participants exhibiting a lack of engagement in vigorous physical activity, the odds of developing ED were 2.26 times higher than in those who were engaged in vigorous physical activity (*p*=0.007, OR=1.25 to 4.10).

Marital status was associated with ED in our univariate analysis; however, there was no statistically significant association after multivariate analysis. Level of education, smoking status, level of income, and engagement in risky sexual behavior did not have a statistically significant association with ED, as shown in **Table 3**.

**Table 3:** Association between sociodemographic factors and erectile dysfunction (*n*=366).

Factors	Erectile dysfunction			
	COR (95% CI)	*p*-value	AOR (95% CI)	*p*-value
Age (years)				
18–39	1		1	
40–59	2.90 (1.71 to 4.90)	0.000	2.91 (1.45 to 5.86)	0.003
60–85	7.27 (3.01 to 17.53)	0.000	7.07 (2.55 to 19.60)	0.000
Marital status				
Married	1		1	
Cohabiting	0.49 (0.43 to 5.48)	0.572	0.49 (0.04 to 5.76)	0.519
Single	0.38 (0.22 to 0.66)	0.924	0.95 (0.45 to 2.00)	0.897
Separated /divorced	0.33 (0.22 to 0.94)	0.090	0.52 (0.24 to 1.11)	0.132
Widow	1.62 (0.46 to 5.7)	0.629	1.37 (0.38 to 4.95)	0.632
Education level				
None	1			
Primary	3.29 (0.99 to 10.92)	0.049	–	–
Secondary	1.58 (0.47 to 5.32)	0.464	–	–
Tertiary	0.67 (0.17 to 2.55)	0.561	–	–
No	1			
Yes	1.35 (0.84 to 2.18)	0.212	–	–
Smoking				
No	1			
Yes	1.62 (0.82 to 3.20)	0.593	–	–
Income				
Low	1			
High	0.56 (0.30 to 1.05)	0.069	–	–
Engage in vigorous physical activity				
Yes	1		1	
No	2.30 (1.30 to 4.06)	0.004	2.26 (1.25 to 4.10)	0.007
Engage in sexual risk behavior				
No	1		–	–
Yes	0.96 (0.60 to 1.53)	0.853		

### Clinical characteristics associated with erectile dysfunction

Depression and reported self-adherence to ART had a statistically significant association with ED in both the univariate and multivariate analyses (*p*< 0.05), whereas anxiety and levels of RBG, testosterone, estradiol, triglyceride, and cholesterol did not have a statistically significant association with ED. The odds of developing ED in participants with mild depression were 2.03 times higher than in participants with no depression (*p*=0.007; 95% CI= 1.21 to 3.41), whereas the odds of developing ED in participants with moderate depression were 3.89 times higher than in participants with no depression (*p*=0.004; 95% CI=1.54 to 9.83). The odds of developing ED in participants with good adherence to ART were 2.79 times higher than in participants with poor adherence to ART (*p*=0.004; OR=1.40 to 5.56) (**Table 4**).

**Table 4:** Association between clinical characteristics and erectile dysfunction.

Factors	Erectile dysfunction			
	COR (95% CI)	*p*-value	AOR (95% CI)	*p*-value
Depression				
No depression	1			
Mild	1.95 (1.17 to 3.24)	0.011	2.03 (1.21 to 3.41)	0.007
Moderate	3.03 (1.25 to 7.36)	0.014	3.89 (1.54 to 9.83)	0.004
Moderate to severe	2.18 (0.58 to 8.14)	0.247	2.77 (0.71 to 10.83)	0.141
Anxiety score				
No anxiety	1			
Mild	1.74 (0.88 to 2.40)	0.148	–	–
Moderate	1.00 (056 to 5.42)	0.336	–	–
Moderate to severe	0.41 (0.06 to 2.99)	0.379	–	
Random blood glucose				
Normal	1			
High	1.65 (0.66 to 4.13)	0.279	–	–
Testosterone level				
Low	1			
Normal	0.76 (0.23 to 2.52)	0.652	–	–
Estradiol levels				
Normal	1			
High	0.81 (0.44 to 1.51)	0.513	–	–
Cholesterol levels				
Normal	1			
High	0.82(0.42 to 1.57)	0.539	–	–
Triglyceride levels				
Normal	1			
High	0.69 (0.40 to 1.20)	0.193	–	–
BMI				
Underweight/normal weight	1			
Overweight/obesity	0.77 (0.48 to 1.30)	0.326	–	–
Hypertension				
No	1			
Yes	1.44 (0.73 to 2.85)	0.294	–	–
ART adherence				
Good	1			
Poor	2.17 (1.41 to 5.19)	0.018	2.79 (1.40 to 5.56)	0.004
Duration of ART				
1–5 years	1			
6–10 years	1.00 (0.56 to 1.79)	1.000	–	–
10 years and above	1.66 (0.93 to 2.95)	0.088		
Type of ART drugs				
1RA	1			
Other types of ART	1.21 (0.47 to 3.09)	0.693	–	–
CD4				
≤200	1			
>200	0.64 (0.29 to 1.43)	0.278	–	–

ART, anti-retroviral therapy; BMI, body mass index; 1RA, tenofovir, lamivudine, and dolutegravir; RBG, random blood glucose.

*1UA, zidovudine, lamivudine, and dolutegravir. 1PA, abacavir, lamivudine, and dolutegravir. 2KA, abacavir, lamivudine, and atazanavir/ritonavir. 2HA, tenofovir, emtricitabine, and atazanavir/ritonavir. 2GA, tenofovir, lamivudine, and efavirenz.

## Discussion

The prevalence of ED among men living with HIV in our study population was high (74.6%). This burden is higher than the prevalence of ED in adult men in the general population in northern Tanzania, which is 29.7% (Nyalile et al, 2020), and it is also higher than that reported in similar studies from Nigeria (39.7%), Belgium (56%), and Spain (61.2%) (17–19). Although these are geographically different contexts, the burden in Tanzania is high and needs to be appropriately addressed. The findings of this study therefore consolidate the evidence that the prevalence of ED is higher in MLHIV and indicate that there is a need for routine screening for erectile dysfunction in MLHIV. This is in line with recommendations from the recent European guideline on the inclusion of sexual dysfunctions, including ED, as comorbidities of HIV (11).

Age was found to be an important risk factor for ED among MLHIV in this study, which was similar to the findings of studies conducted in Nigeria, Barcelona, and West Malaysia (17, 20, 21). The prevalence of ED increases with advancing age as underlying risk factors such as vascular diseases, hypertension, diabetes mellitus, and obesity are more common in the elderly population (22). Engagement in vigorous physical activity was also found to be associated with ED among MLHIV in Tanzania. The relationship between intense physical activity and enhanced sexual performance has been well documented in systematic reviews and randomized clinical trials (22, 23). There is a growing body of evidence showing that the health benefits of engaging in intense physical activity are similar for the general population and people living with HIV (23). Therefore, this study provides evidence for the role of intense physical activity in the primary prevention of ED for people living with HIV, as in the general population.

Depression was present in 66.7% of MLHIV, whereas anxiety was present in 48.4% of MLHIV. Depression and anxiety both significantly interfere with an individual’s physical, psychological and social function (6, 8). However, only depression was found to be significantly associated with ED in a multivariate analysis. This was similar to findings from studies in Europe, Malaysia, and Iran, which found that patients with ED had a higher rate of depression (17%) than those without ED (6, 7, 20). Depression is the most common neuropsychiatric condition associated with HIV, and the effective treatment of both depression and erectile dysfunction has been reported to improve the quality of life in MLHIV (23–25).

Self-reported adherence to ART was also associated with ED among MLHIV in Tanzania, which was similar to findings in studies carried out in the USA (6, 12). Long-term adherence to ART is essential for sustained viral suppression to an undetectable level; therefore, there is a need for concurrent assessment and treatment of ED and HIV to be conducted for people enrolled in CTC clinics (9). The introduction of ART has been shown to decrease the prevalence of hypogonadism, which was more common before its introduction (9). However, almost all types of ART are associated with some degree of ED; and this is most significant with protease inhibitors (8, 9, 11, 12).

The findings of this study have shown no statistically significant association between types of ART and ED, contrary to those of other studies. This could be due to the fact that most of the participants were receiving first-line ART (92.6%). In addition, there was no association between the duration of ART and ED. This is similar to the findings of a study carried out by De Vicentis et al. (11), although is in contrast to those of other studies, which show an association between the duration of ART and ED (7). The findings of this study also contrast with those of a study conducted by Crum-Cianflone et al., which showed that a higher CD4 count was protective against ED (7).This difference could be accounted for by the variation in the clinical characteristics of study participants, such as ethnicity, and the prevalence of hypogonadism.

Furthermore, this study shows that there is a higher prevalence of hypogonadism in MLHIV (92.7%). Several studies have also shown that there is an association between HIV and low testosterone (6, 7, 11). However, there was no statistically significant association between low testosterone levels and ED, probably because most of the participants had low testosterone. HIV can cause low testosterone, although currently, the relationship between HIV and testosterone is a controversial one, as other studies have found normal testosterone levels in men living with HIV (25, 26).

Dyslipidemia was not associated with ED in MLHIV in this study, contrary to evidence from a study in Mexico (10). This could be due to the difference in clinical characteristics between the study groups, as there was a low prevalence of dyslipidemia among our study participants.

The factors assessed in our study, including increased blood glucose level, hypertension, BMI, alcohol use, and cigarette smoking, were not associated with ED, which is a finding similar to that of a study conducted in the USA (22). However, other studies have shown that there is an association between the above variables and ED (7). This could be due to differences in population characteristics between this study and other studies.

Contrary to other studies (6, 12), this study found that there was no association between ED and risky sexual behaviors. This could be due to differences in the characteristics of study participants, such as sexual orientation. However, the number of MLHIV who are engaged in risky sexual behavior is alarming (44.6%).

### Strengths and limitations

This was a multi-center study, allowing the standardization of laboratory investigations conducted to examine the organic causes of ED. It also assessed organic and psychological factors simultaneously. However, the convenience sampling technique was used in this study, meaning that the study has poor generalizability and making it prone to estimate bias.

## Conclusion

There was a high prevalence of ED among MLHIV in northern Tanzania. Erectile dysfunction should be regarded as one of the comorbidities of HIV and should be routinely screened for, alongside evidence-based interventions among MLHIV in CTC clinics.

## Ethical approval

Ethical approval was obtained from Kilimanjaro Christian Medical University College (KCMUCo) institution ethical review board (certificate number 2547). Permission to conduct the study was sought from the District Medical Officer of Moshi Municipality, the medical officer in charge of Mawenzi hospital, and the Executive Director of KCMC. All study participants were given a written informed consent form prior to participating in the interview.

## Data availability statement

The raw data supporting the conclusions of this article will be made available by the authors, without undue reservation.

## Ethics statement

The studies involving humans were approved by Kilimanjaro Christian Medical College Research Ethics and Review Committee. The studies were conducted in accordance with the local legislation and institutional requirements. The participants provided their written informed consent to participate in this study.

## Author contributions

OM conceptualized the idea. OM, ML, KA, GN, and PG participated in data collection and writing the first draft. OM and EM participated in data analysis. BN, EM, MA, JN, EM, GK, RM, BK, SM, EL, BS, JB, BM, and AM participated in mentoring and supervision of research, proposal development, and report writing. All authors contributed to the article and approved the submitted version.
